# Gender-affirming hormone therapy, quality of life, and the role of oestradiol and testosterone in transgender individuals

**DOI:** 10.1017/neu.2025.10018

**Published:** 2025-06-05

**Authors:** Emma Elisabeth Skovby Petersen, Frederika Kiy, Ulrik Schiøler Kesmodel, Maria Lucia Pop, Gry Kjaersdam Telléus, Allan Stensballe, Jakob Dal, Astrid Højgaard, Michael Winterdahl

**Affiliations:** 1 Sexological Center, Aalborg University Hospital, Stengade 10, Stuen 9000Aalborg, Denmark; 2 Department of Obstetrics and Gynaecology, Aalborg University Hospital, Aalborg, Denmark; 3 Psychiatry, Aalborg University Hospital, Aalborg, Denmark; 4 Department of Communication and Psychology, Aalborg University, Aalborg, Denmark; 5 Translational Pain Neuroscience and Precision Health, Department of Health Science and Technology, Aalborg University, Aalborg, Denmark; 6 Clinical Cancer Research Unit, Aalborg University Hospital, Aalborg, Denmark; 7 Department of Endocrinology, Aalborg University Hospital, Aalborg, Denmark; 8 Steno Diabetes Center North Jutland, Aalborg, Denmark; 9 Translational Neuropsychiatry Unit, Department of Clinical Medicine, Aarhus University, Aarhus, Denmark

**Keywords:** Quality of life, transgender persons, gonadal steroid hormones, oestradiol, testosterone

## Abstract

**Objectives:**

The present study examines the quality of life (QoL) of transgender and gender-diverse individuals receiving versus not receiving gender-affirming hormone therapy (GAHT) in those assigned male at birth (AMAB) and assigned female at birth (AFAB). It also explores the relationship between QoL and concentrations of oestradiol and testosterone.

**Methods:**

This cross-sectional study used the WHOQOL-BREF questionnaire to assess QoL. Participants were categorised into four groups based on assigned sex at birth (AMAB or AFAB) and GAHT status, with non-GAHT participants serving as controls. MANOVA and t-tests were used to compare QoL between groups, and linear regression analyses examined associations between QoL and oestradiol/testosterone concentrations in AMAB and AFAB participants.

**Results:**

The study included 360 participants: 169 AMAB (143 receiving GAHT and 26 controls) and 191 AFAB (141 receiving GAHT and 50 controls). GAHT recipients had significantly higher QoL than controls in both AMAB (p < 0.01) and AFAB (p = 0.02) groups, particularly in the psychological health domain (D2). AFAB participants reported higher overall QoL than AMAB in both GAHT (p = 0.01) and control (p = 0.04) groups, with significance in the social relationship domain among GAHT participants. No significant relationship was found between oestradiol concentrations and QoL for participants AMAB. However, a significant relationship between testosterone concentrations and QoL was observed only in the social relationship domain (D3) for participant AFAB.

**Conclusion:**

This study highlights the benefits of GAHT for QoL and differences in QoL between individuals AMAB and AFAB.

## Graphical abstract

**Figure:**
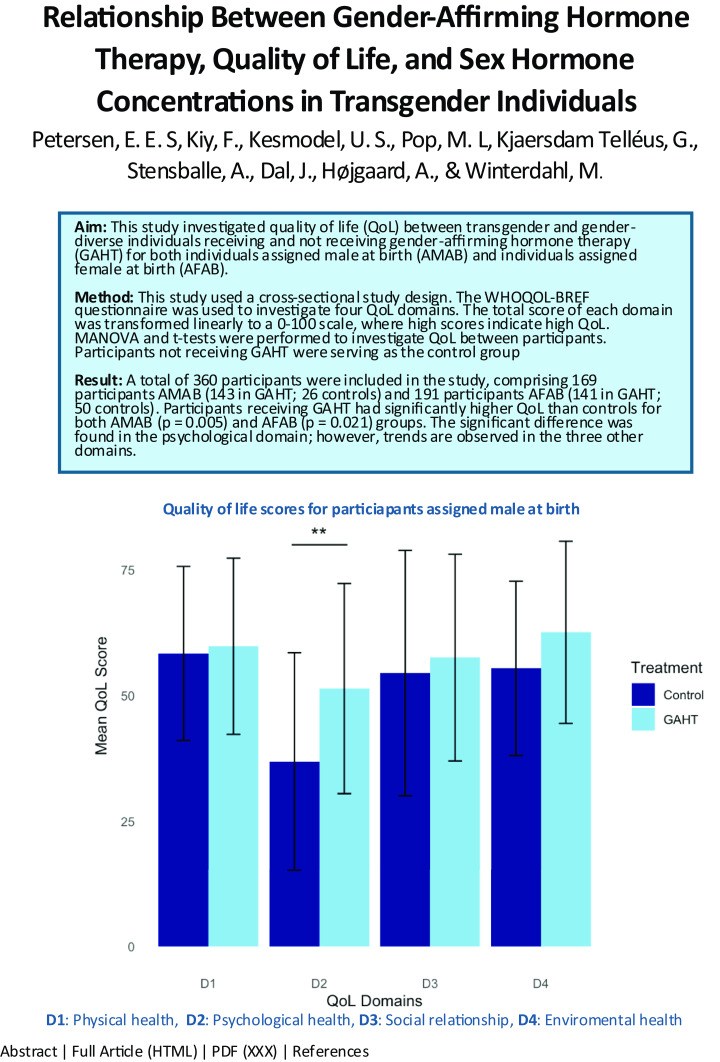

Significant outcomes

- Participants receiving GAHT had a statistically significantly higher QoL compared to controls for both participants AMAB and participants AFAB
- The significant difference in QoL between assigned gender at birth groups, highlights the importance of distinguishing between individuals AMAB and AFAB in future studies
- A significant positive linear relationship was found between testosterone concentrations and the social relationship QoL domain (D3) for participants AFAB.

Limitations

- Small Subgroup Sizes: The design divided the 360 TGD participants into smaller subgroups, limiting the power to conduct sub- and confounder-adjusted analyses, which may affect the robustness of the findings.
- Potential Bias in WHOQOL-BREF Responses: The reliance on the WHOQOL-BREF questionnaire, which involves subjective interpretation, could impact internal validity as participants might interpret questions differently, potentially influencing the QoL assessments.
- Timing and Consistency of Hormone Measurements: There was a time difference of up to one month between blood tests and questionnaire responses, and variability in the timing of blood sampling relative to GAHT administration, which could affect the accuracy of the relationship between oestradiol/testosterone concentrations and QoL.

## Introduction

Transgender and gender-diverse (TGD) individuals are terms used to describe people whose gender identity does not align with their assigned sex at birth (Rajkovic *et al*., 2022). The prevalence of TGD individuals is estimated to be 0.54–1.1% of the world’s population (Arcelus *et al*., 2015; Van Caenegem *et al*., 2015; Frisch, 2019; Herman et. al. 2022). Transgender women are individuals identifying themselves as women but assigned male at birth (AMAB) and transgender men are individuals who identify as men but assigned female at birth (AFAB) (Frisch *et al*., 2019; T’Sjoen *et al*., 2019). Individuals whose gender is neither woman nor man often describe themselves as nonbinary (T’Sjoen *et al*., 2019).

Over the past decades the number of referrals of TGD individuals to gender-affirming hormone treatment (GAHT) has increased (Delahunt *et al*., 2018; Leinung & Joseph, 2020; Glintborg *et al*., 2022; McKechnie *et al*., 2023). The purpose of GAHT is to relieve possible gender incongruence by reducing and replacing endogenous sex hormones. This will lead to a change in the physical appearance that will make it more congruent with the individual’s gender identity (Hembree *et al*., 2017; Foster Skewis *et al*., 2021). Feminising GAHT consists of oestrogen and anti-androgens, which among others will induce breast development, reduction in facial and body hair, and redistribution of fat. In contrast, masculinising GAHT includes the administration of testosterone where the most important effects are deepening of the voice, increase in muscle mass, increase in facial and body hair, and cessation of menstruation (T’Sjoen *et al*., 2019).

The physiological changes associated with GAHT are believed to enhance quality of life (QoL) by enabling individuals ‘to live and be accepted as a person of the experienced gender’ leading to positive effects on various aspects of psychological well-being (Gorin-Lazard *et al*., 2013; World Health Organization (WHO), 2019/2021; T’Sjoen *et al*., 2019; Silva *et al*., 2021). Several studies align with these observations, suggesting that GAHT may improve the QoL of TGD individuals (Nguyen *et al*., 2018; Rowniak *et al*., 2019; Meyer *et al*., 2020; Baker *et al*., 2021). Additionally, a recent randomised controlled trial demonstrated improved self-rated health among TGD individuals in masculinising GAHT (Nolan *et al*., 2024). However, findings on this topic remain inconsistent, with some studies reporting no significant QoL improvements following GAHT (Pavanello Decaro *et al*., 2021; Filipov *et al*., 2023).

QoL is influenced by several psychological factors, including fatigue, stress, behaviour, and mood (Salehpoor *et al*., 2014; Rieu *et al*., 2016; Zitzmann, 2020). It is well known that sex hormones, such as testosterone or oestradiol, can influence brain functions by modulating neurotransmitters and thereby regulating mental factors (Giltay *et al*., 2008; Barth *et al*., 2015). A study by Giltay et al., has shown that feminising GAHT may have negative effects on neurotransmitter mediated mood, behaviour, fatigue, and stress-coping, whereas masculinising GAHT has beneficial effects on the same mental factors (Giltay *et al*., 2008). Since sex hormones affect the physiological function of the brain and thereby also mental wellbeing, it suggests a potential relationship between sex hormone concentrations and QoL. However, only limited evidence exists in this field, with insufficient research on the relationship between GAHT and QoL.

Therefore, this study aimed to investigate the relationship between GAHT and QoL as well as exploring how testosterone concentrations in AFAB individuals and oestradiol concentrations in AMAB individuals impact QoL.

## Methods

### Study participants

Transgender and gender-diverse (TGD) individuals referred to the Centre for Gender Identity (CGI), Aalborg University Hospital, Denmark, were recruited for this study. Eligibility was determined using contact codes DZ768E1-E4, which are used in the Danish healthcare system for gender identity-related cases. Participants were invited via the public Danish digital mailbox, e-Boks, to join the TraCK (Transkohorte ved Center for Kønsidentitet) cohort, from which study data were derived. Study participants were enrolled between February 14 and May 6, 2024. Both TGD individuals referred to CGI for medical evaluation and TGD individuals receiving GAHT were included in this study. At CGI, GAHT is provided by a multidisciplinary team including psychologists, psychiatrists, gynaecologists, endocrinologists, who collectively determine whether there is an indication for treatment after comprehensive psychological and medical evaluation.

### Inclusion criteria

Participants were eligible for inclusion if they provided informed consent, completed the WHOQOL-BREF questionnaire (Bech & Nørholm, 2001), and were proficient in Danish, as the questionnaire was only available in Danish. Additional inclusion criteria specifically for the third research question were that participants must have undergone GAHT and received a blood test measuring oestradiol concentration for those AMAB and testosterone concentrations for those AFAB, within seven days to one month of completing the questionnaire. Another inclusion criterion was that participants in the GAHT group must have received GAHT for at least three months, whereas those in the control group must not have received any GAHT, either previously or within the last six months including self-medication.

### Study design

This study used a cross-sectional study design. Participants were divided into four groups based on their assigned sex at birth and whether they received GAHT. The four groups consisted of: participants AMAB who received GAHT, participants AMAB not receiving GAHT (control group), participants AFAB who received GAHT, and participants AFAB not receiving GAHT (control group).

### Data collection

Data were collected from TraCK which contains information about TGD individuals from CGI, Aalborg. The database was created in Research Electronic Data Capture (REDCap), which is a secure, web-based software platform designed to support data capture for research studies (Harris *et al*., 2009, 2019). Personal information was managed in compliance with General Data Protection Regulation and the National Data Protection Act and was registered in North Denmark Region’s internal directory, ID-number F2024-012.

### Outcome measures

Participants enrolled in this study completed the Danish version of the WHOQOL-BREF questionnaire (Bech & Nørholm, 2001). The WHOQOL-BREF is a validated questionnaire consisting of 26-items distributed across four domains: physical health (D1), psychological health (D2), social relationships (D3), and environmental health (D4) (with 7, 6, 3, and 8 questions, respectively) (Bech & Nørholm, 2001; Vahedi, 2010). The remaining two questions are related to general health; however, these are not included in this study. Each item is scored from 1 to 5, and the total score of each domain is transformed linearly to a 0-100-scale, where high scores indicate high QoL (University of Washington Seattle and Oliver, 1997). Missing data for a single question was managed by utilising the average score from the other questions within the same domain to calculate the missing score (Programme on mental health, 1996). Blood sample results were collected from medical records stored in the TraCK database. The blood test included plasma oestradiol from participants AMAB, and plasma testosterone from participants AFAB and were utilised for subsequent analyses. The blood samples used in this study are part of routine tests conducted during participant’s medical checkups at CGI. TGD individuals who have recently initiated GAHT undergo blood samples one month after initiating GAHT and thereafter every three months during the first year of treatment, while the other TGD individuals in GAHT have blood samples taken half-yearly to annually as part of their routine medical checkups. Samples were collected at participants’ local laboratories, resulting in variations in collection times and analysis.

### Data analysis

Before conducting the statistical analysis, a statistical analysis plan was developed to ensure the transparency and reproducibility of this study. Data were analysed using R (version 4.3.3; R Core Team, 2024). To test for normality of the dependent variables skewness and kurtosis were calculated, and to test the homoscedasticity Levene’s test were performed. Based on the distribution, data were presented with means (±SD).

Multivariate analysis of variance (MANOVA) was conducted to examine differences in the four QoL domains between AMAB participants receiving GAHT and their control group, as well as between AFAB participants receiving GAHT and their control group. Additionally, MANOVA was used to assess differences in QoL domains between AMAB and AFAB participants receiving GAHT and their respective controls. To further explore group differences, two-sided t-tests were performed for each QoL domain, comparing treatment versus control groups and AMAB versus AFAB groups.

Linear regression analyses were performed to investigate the relationship between hormone concentrations of plasma oestradiol (pmol/L) (continuous) and plasma testosterone (nmol/L) (continuous), and the four QoL domains (continuous) for participants AMAB and participants AFAB, respectively. These linear regression analyses were performed for both those who had a blood test within seven days and those who had a blood test within one month of answering the WHOQOL-BREF questionnaire.

Skewness and Kurtosis were calculated to test the normality of the residuals, and log transformation of the independent variables was performed. For each regression analysis, R^2^ was calculated to investigate the amount of variation in the dependent variable that can be explained by the independent variable. Furthermore, regression coefficients and 95% confidence intervals (95% CI) were calculated for each regression analysis. The level of significance was set at 0.05. No attempt was made to adjust for confounding because of small sample size.

## Results

Data from 424 participants were collected for this study. Sixty-four participants were excluded from this study because they did not fulfil the inclusion criteria (45 participants due to incomplete responses to the WHOQOL-BREF questionnaire, 16 participants from the GAHT group due to receiving GAHT less than three months, and three participants from the control group due to receiving GAHT within the last six months), thus leaving 360 participants for further data analysis. The average duration of GAHT for participants AMAB was 45.5 months, with a range from 3 to 410 months, and the average duration of GAHT for participants AFAB was 48.6 months, with a range from 3 to 303 months.

### Demographics

Of the 360 participants, 169 participants were AMAB (143 receiving GAHT and 26 controls), and 191 participants were AFAB (141 receiving GAHT and 50 controls). Demographics regarding age, gender identity, education, ethnicity, and occupation are shown in Table 1.

**Table 1.** Demographics of the 360 participants included in the study

Group	GAHT (AMAB)	Control (AMAB)	GAHT (AFAB)	Control (AFAB)
Total (n)	143	26	141	50
Age (Mean(SD))	33 (14)	28 (10)	28 (8)	25 (7)
	n (%)	n (%)	n (%)	n (%)
Gender Identity*				
Man	0 (0)	0 (0)	98 (70)	28 (56)
Woman	125 (87)	19 (73)	0 (0)	0 (0)
Non-binary	19 (13)	6 (23)	35 (25)	17 (34)
Queer	16 (11)	5 (19)	28 (20)	6 (12)
Other gender identity^a^	40 (28)	12 (46)	58 (41)	20 (40)
**Education**				
Lower secondary school	14 (10)	3 (12)	15 (11)	6 (12)
10^th^ grade	8 (6)	5 (19)	24 (17)	13 (26)
Vocational education	17 (12)	2 (8)	12 (9)	2 (4)
High school	46 (32)	9 (35)	42 (30)	19 (38)
Short-cycle higher education	9 (6)	1 (4)	7 (5)	2 (4)
Medium-cycle higher education	24 (17)	4 (15)	25 (18)	3 (6)
Long-cycle higher education	19 (13)	2 (8)	13 (9)	2 (4)
Other^b^	6 (4)	0 (0)	3 (2)	3 (6)
**Ethnicity**				
Danish	123 (86)	20 (77)	112 (79)	39 (78)
Other^c^	20 (14)	6 (23)	29 (21)	11 (22)
**Occupation**				
Employed	47 (33)	6 (23)	43 (30)	7 (14)
Unemployed	6 (4)	2 (8)	8 (6)	1 (2)
Enrolled in education	33 (23)	11 (42)	41 (29)	22 (44)
Financial assistance	20 (14)	1 (4)	23 (16)	7 (14)
Early retirement pension	14 (10)	1 (4)	9 (6)	4 (8)
Other^d^	23 (16)	5 (19)	17 (12)	8 (16)

SD: standard deviation. Percentages represent the portion of participants in each group.

AMAB: assigned male at birth. AFAB: assigned female at birth.

* n may exceed the total number of participants in each group as it is possible to have multiple gender identities. This was chosen to account for the fluidity of gender identity, which can change over days or weeks.

a Both man and woman, neither man nor woman, predominantly man, predominantly women, agender, gender-fluid, don’t know, unsure.

b Not completed lower secondary school, specially planned youth education (STU).

c Faroese, Polish, Indian, half Greek, Icelander, Slovaks, Greenlander, German, half Moroccan, Norwegian, South African, English, French, Venezuelan, American, Roman, Italian, Spanish, Thai, half Iranian, Russian, Singaporean, Swiss, Korean, half Turkish, Finnish, Bosnian, Chinese, Australian, Kurdish, Dutch, no answer.

d Flex job, state pension, sickness benefits, living of own savings, supported by a partner, supported by family, supported by a friend.

### Relationship between gender-affirming hormone therapy and quality of life

**Figure 1.**
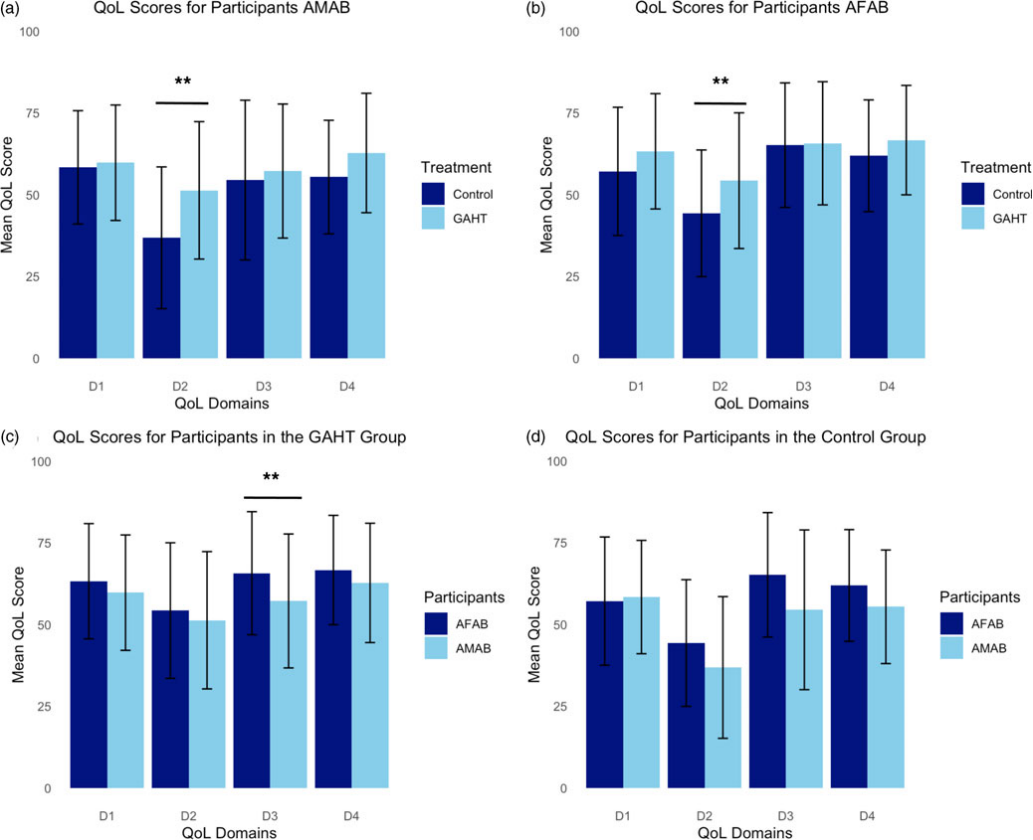
Bar charts illustrating mean quality of life (QoL) scores on the *y*-axis for each QoL domain (D1: physical health; D2: psychological health; D3: social relationships; D4: environmental health) on the *x*-axis. (a) QoL mean scores for gender-affirming hormone therapy (GAHT) and control groups for participants assigned male at birth (AMAB) (*n* = 143; *n* = 26, respectively). (b) QoL mean scores for GAHT and control groups for participants assigned female at birth (AFAB) (*n* = 141; *n* = 50, respectively). (c) QoL mean scores for participants AMAB and participants AFAB in the GAHT group (*n* = 143; *n* = 141, respectively). (d) QoL mean scores for participants AMAB and participants AFAB in the control group (*n* = 26; *n* = 50, respectively). Standard deviations are illustrated as error bars. Significant differences in each domain are marked with ** indicating a significance level of less than 0.01.

The result of the MANOVA showed statistically significant higher mean QoL scores in the GAHT group compared to the control group for participants AMAB (*p* < 0.01) (Fig. 1a). However, this significant difference was primarily driven by the psychological health domain (D2) where post hoc analysis revealed that a statistically significant higher mean QoL score was found for the GAHT group (51.4) compared to the control group (36.9) for participants AMAB (*p* < 0.01). No statistically significant difference in mean QoL scores for the other three QoL domain scores (physical health (D1), social relationships (D3), and environmental health (D4)) between the GAHT- and the control group was found for participants AMAB (*p* = 0.70; *p* = 0.55; *p* = 0.06, respectively)

For participants AFAB the result of the MANOVA also showed statistically significant higher mean QoL scores for the GAHT group compared to the control group (*p* = 0.02) (Fig. 1b). However, this significant difference was primarily driven by the psychological health domain (D2), where post hoc analysis revealed that a statistically significant higher mean score for the GAHT group (53.9) compared to the control group (44.3) was found for participants AFAB (*p* < 0.01). No statistically significant difference in mean QoL scores of the other three QoL domains (physical health (D1), social relationships (D3), and environmental health (D4)) was found for participants AFAB (*p* = 0.07, *p* = 0.95; *p* = 0.10, respectively).

### Relationship between assigned sex at birth and quality of life

The results of the MANOVA showed a statistically significantly higher mean QoL scores for participants AFAB compared to participants AMAB in the GAHT group (*p* = 0.01) (Fig. 1c). However, this significant difference was primarily driven by the social relationship domain, where post hoc analysis revealed a higher mean QoL score for participants AFAB (65.4) compared to participants AMAB (57.5) (*p* < 0.01) for those receiving GAHT. There was no significant difference in mean QoL scores of the three remaining QoL domains (physical health (D1), psychological health (D2), and environmental health (D4)) for those receiving GAHT between participants AFAB and participants AMAB (*p* = 0.13; *p* = 0.31; *p* = 0.05, respectively). Furthermore, the results of the MANOVA showed statistically significantly higher mean QoL scores for participants AFAB compared to participants AMAB in the control group (*p* = 0.04) (Fig. 1d). Nevertheless, the post hoc analysis found no significant difference in the control group in the four specific QoL domains between participants AFAB and participants AMAB (*p* = 0.78; *p* = 0.15; *p* = 0.06; *p* = 0.13, respectively).

### Relationship between oestradiol concentrations and quality of life

**Figure 2.**
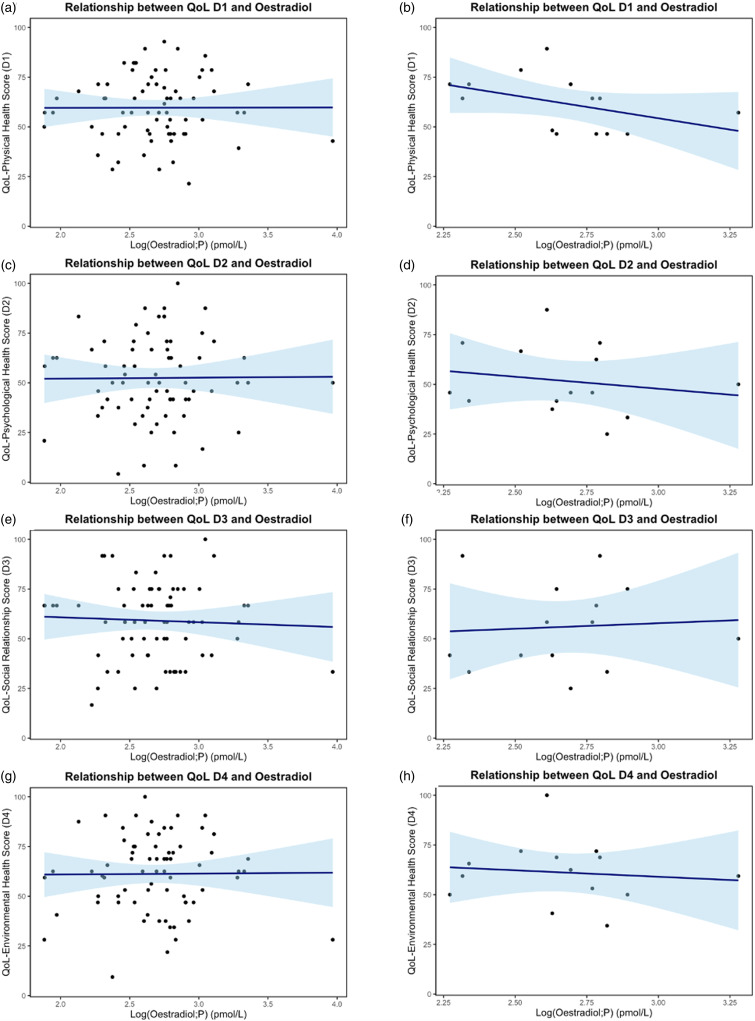
Relationship between oestradiol concentrations and the four quality of life (QoL) domain scores for participants assigned male at birth, (a) and (b) D1: physical health, (c) and (d) D2: psychological health, (e) and (f) D3: social relationship, (g) and (h) D4: environmental health. The y-axes represent the QoL domain scores and the x-axes represent log (Oestradiol; P) in pmol/L. Confidence intervals are illustrated with a light blue colour. Figure a, c, e, and g: *n* = 71, one moths interval between measurements. Figure b, d, f, and h: *n* = 14, seven days interval between measurements.

A total of 71 AMAB participants were included in the analyses, with a maximum interval of one month between the two measurements: QoL score and oestradiol concentration. Additionally, 14 AMAB participants were analysed with a maximum interval of seven days between measurements. The median oestradiol concentration was 495 pmol/L (lower quartile: 308 pmol/L; upper quartile: 691 pmol/L) for participants with a 1-month interval, and 467 pmol/L (lower quartile: 349 pmol/L; upper quartile: 620 pmol/L) for those with a 7-day interval. Regression coefficients, 95% CI, and *R* ^2^-values are shown in Table 2 for both time intervals. Figure 2 illustrates the linear regression plots representing the relationship between oestradiol concentrations and the four QoL domain scores for both time intervals. No significant linear relationship was found between oestradiol concentrations and the four QoL domain scores.

**Table 2.** Relationship between oestradiol concentrations and quality of life (QoL) domain scores for participants assigned male at birth

QoL Domain	a	b	Lower 95% CI	Upper 95% CI	R^2^
**D1**					
Max. one month	59.35	0.1	−10.88	11.09	−0.014
Max. seven days	122.76	−22.82	−52.5	6.87	0.122
**D2**					
Max. one month	51.1	0.49	−13.5	14.47	−0.014
Max. seven days	83.91	−12.04	−52.72	28.65	−0.047
**D3**					
Max. one month	65.66	−2.45	−15.57	10.68	−0.012
Max. seven days	41.06	5.58	−45.67	56.83	−0.078
**D4**					
Max. one month	60.02	0.45	−12.48	13.38	−0.014
Max. seven days	78.53	−6.51	−44.48	31.47	−0.071

Regression coefficients, lower and upper 95% confidence intervals (CI), and R^2^ for each QoL domain (D1: physical health; D2: psychological health; D3: social relationships; D4: environmental health) are illustrated for both maximum (max) one month between the measurements and seven days between the measurements. *n* = 71.

^a^Intercept.

^b^Slope.

### Relationship between testosterone concentrations and quality of life

**Figure 3.**
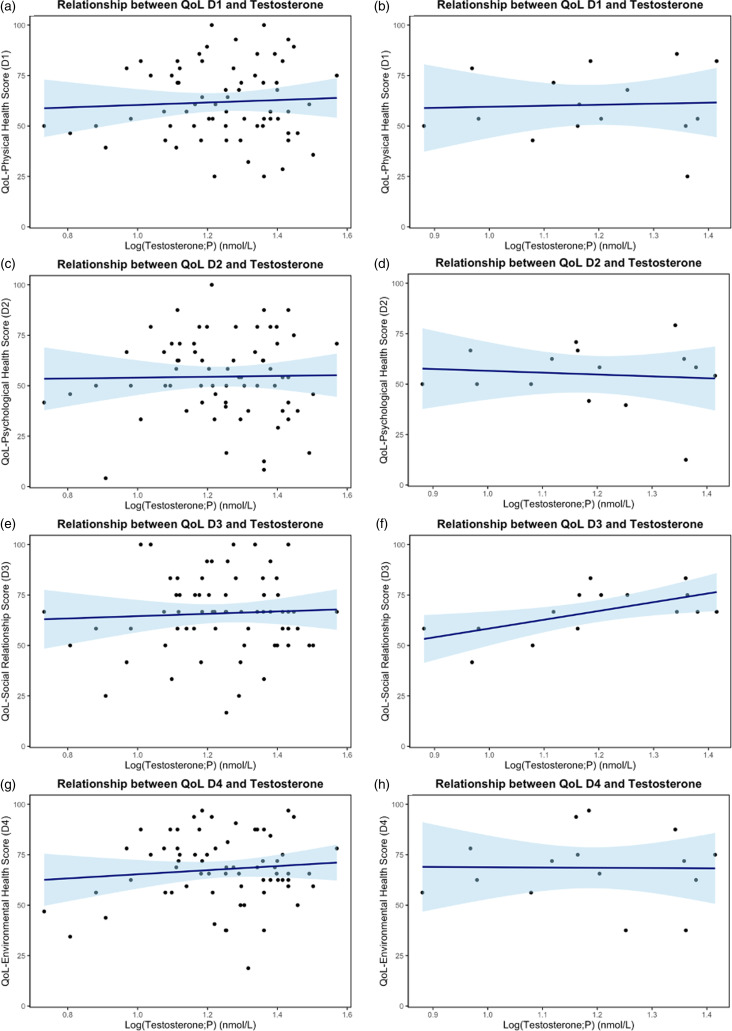
Relationship between testosterone concentrations and the four quality of life (QoL) domain scores for participants assigned female at birth, (a) D1: physical health, (b) D2: psychological health, (c) D3: social relationships, (d) D4: environmental health. The y-axes represent the QoL domain scores and the x-axes represent log(Testosterone; P) in nmol/L. Confidence intervals are illustrated with a light blue colour. Figure a, c, e, and g: *n* = 67, one moths interval between measurements. Figure b, d, f, and h: *n* = 14, seven days interval between measurements.

A total of 67 AFAB participants were included in the analyses, with a maximum interval of one month between the two measurements: QoL score and testosterone concentration (two were excluded due to a testosterone concentration >52 nmol/L). Additionally, 15 AFAB participants were analysed with a maximum interval of seven days between measurements. The median testosterone concentration was 17 nmol/L (lower quartile: 12 nmol/L; upper quartile: 23 nmol/L) for participants with a 1-month interval, and 14 nmol/L (lower quartile: 12 nmol/L; upper quartile: 21 nmol/L) for those with a 7-day interval. Regression coefficients, 95% CI, and R^2^-values are illustrated in Table 3 for both time intervals. Figure 3 illustrates the linear regression plots representing the relationship between testosterone concentrations and the four QoL domain scores for both time intervals. A significant linear relationship was found between testosterone concentrations and the social relationships QoL domain (D3) (*p* = 0.02) among participants within the seven-day interval. No significant linear relationship was found between testosterone concentrations and the QoL domain scores for physical health (D1), psychological health (D2), and environmental health (D4). Furthermore, no significant linear relationship was found between testosterone concentrations and the four QoL domain scores among participants within the one-month interval.

**Table 3.** Relationship between testosterone concentrations and quality of life (QoL) domain scores for participants assigned female at birth

QoL Domain	a	b	Lower 95% CI	Upper 95% CI	R^2^
**D1**					
Max. one month	54.34	6.08	−20.47	32.62	−0.012
Max. seven days	54.45	5.07	−57.27	67.41	−0.074
**D2**					
Max. one month	51.84	2.15	−26.87	31.16	−0.015
Max. seven days	65.77	−9.16	−66.75	48.43	−0.067
**D3**					
Max. one month	58.75	5.77	−21.34	32.9	−0.013
Max. seven days	14.88	43.51	9.47	77.56	0.321
**D4**					
Max. one Month	55.12	10.18	−13.84	34.2	−0.004
Max. seven days	70.17	−1.37	−65.18	62.44	−0.077

Regression coefficients, lower and upper 95% confidence intervals (CI), and R^2^ for each QoL domain (D1: physical health; D2: psychological health; D3: social relationships; D4: environmental health) are illustrated for both maximum (max.) one month between the measurements and seven days between the measurements. *n* = 67.

aIntercept.

bSlope.

## Discussion

Our study shows that TGD individuals receiving GAHT report significantly higher QoL scores compared to those not receiving treatment, particularly in the psychological health domain (D2). Moreover, AFAB participants reported higher scores in the social relationship domain (D3) compared to AMAB participants, suggesting possible gender-specific differences – either in the effects of GAHT, or in patterns of social integration and support. Additionally, when testosterone measurements were taken within a short interval of seven days of the QoL assessment, a linear relationship emerged between testosterone concentrations and QoL, highlighting the potential mood-enhancing effects of testosterone. However, the absence of a consistent relationship across all longer intervals and QoL dimensions once again suggests that factors beyond hormonal levels – such as social affirmation, psychological resilience and access to support – play a crucial role in overall well-being. Our findings highlight the importance of gender-affirming healthcare in improving well-being among TGD individuals while also underscoring the complexity of factors influencing QoL.

### Gender-affirming hormone therapy and quality of life

The observed improvements in QoL following GAHT are consistent with previous studies (Baker *et al*., 2021; van Leerdam *et al*., 2023), reinforcing the potential role of medical transition in supporting mental health and well-being among TGD individuals. However, our findings suggest that the effect of GAHT may not be uniformly distributed across all domains of QoL. Specifically, the improvements were primarily observed in the psychological health domain (D2), with no statistically significant differences in the physical health (D1), social relationships (D3), or environmental health (D4) domains. This pattern implies that, rather than through broader improvements in daily functioning or social conditions, GAHT may exert its benefits through psychological mechanisms – such as the alleviation of gender dysphoria or improvement of gender congruence, the enhancement of self-esteem and overall subjective wellbeing. In addition, other studies have also reported reduction in anxiety and depression following GAHT (Gómez-Gil *et al*., 2014; Heylens *et al*., 2014). This study also supports results from the recent randomised controlled trial by Nolan *et al*. (2024), which showed improved self-rated health following masculinising GAHT.

In contrast, some studies have reported mixed or null findings regarding GAHT and QoL (Simbar *et al*., 2018; Foster Skewis *et al*., 2021; Filipov *et al*., 2023). Methodological differences may explain these discrepancies. For instance, Simbar *et al*. (2018) assessed QoL as a single global score, which could have masked domain-specific effects. Foster Skewis *et al*. (2021) only followed individuals for six months post-GAHT initiation, which may not be sufficient to observe significant changes in QoL. Moreover, studies that reported no improvement often included participants using self-prescribed GAHT (Doyle *et al*., 2023; van Leerdam *et al*., 2023), which may introduce additional stressors related to inconsistent dosing, lack of medical supervision, and healthcare barriers.

The study by Filipov et al., did not find a difference in QoL for individuals AFAB following GAHT compared to those who did not receive GAHT which could be due to the small sample size (Filipov *et al*., 2023). In addition, the study by Foster Skewis et al., did not find a difference in QoL for individuals AMAB after initiating GAHT (Foster Skewis *et al*., 2021). Notably, the study by Foster Skewis et al., differs from our study and the other two studies in terms of study design and the QoL assessment tool utilised, as the study by Foster Skewis et al., is a prospective cohort study and uses the RAND SF-36 questionnaire (Simbar *et al*., 2018; Foster Skewis *et al*., 2021; Filipov *et al*., 2023). It is important to note that the longitudinal findings from the prospective cohort study by Foster-Skewis et al., are methodologically more reliable compared to our study and other cross-sectional studies. Furthermore, it is not yet clear which QoL assessment tool is the most appropriate for transgender individuals, which could also be a reason for differences in findings between studies.

### Differences in QoL between AMAB and AFAB participants

A study by Gorin-Lazard et al., in 2012 also found a significant improvement in the social and psychological QoL dimension for AMAB individuals and AFAB individuals receiving GAHT, however, they did not find a difference in QoL between individuals AMAB and AFAB following GAHT (Gorin-Lazard *et al*., 2012). This finding is in contrast to our study, where a significantly higher QoL score in the social relationship domain(D3) was found for AFAB participants compared to AMAB participants receiving GAHT. An explanation for this could be the larger sample size of our study compared to the sample size of Gorin-Lazard et al., (AMAB: *n* = 31; AFAB: *n* = 30) (Gorin-Lazard *et al*., 2012).

The significant difference found in our study could also be explained by the faster onset of physical changes following masculinising GAHT, and more comprehensive results compared to feminising GAHT. For example, individuals AFAB experience a change in voice three months after initiating GAHT, whereas a change in voice is not possible for individuals with AMAB if GAHT is initiated after puberty, which can impact social relations (Bultynck *et al*., 2017). In addition, individuals AFAB get faster access to top surgery (mastectomy), min. 6 months after initiating GAHT, compared to a limit of at least 12 to 24 months for breast augmentations for individuals AMAB (Danish Health Authority, 2023). Altogether, these factors can influence body congruence which may affect the way individuals AMAB and individuals AFAB respond to and are perceived in social settings.

Furthermore, testosterone may exert more immediate behavioural effects, potentially contributing to increased confidence, assertiveness, and social engagement. This may occur through central neurobiological mechanisms, as sex steroids can cross the blood–brain barrier and bind to androgen and oestrogen receptors in brain regions involved in emotion regulation, social behaviour, and reward processing – such as the amygdala, hippocampus, and prefrontal cortex (Giltay *et al*., 2008; Barth *et al*., 2015). These central effects may further enhance social functioning in AFAB individuals during early stages of GAHT.

Finally, sociocultural factors must also be considered. A qualitative study by study by Verbeek *et al*., from (2020) found that transgender women experience higher levels of social stigma and gender-based discrimination than transgender men, which also could be an explanation for the significant difference observed in the social relationship domain (D3) between individuals AMAB and AFAB (Verbeek *et al*., 2020). These combined biological, psychological, and social dynamics may help explain the significantly higher QoL scores in the social relationship domain (D3) observed for AFAB participants in our study.

### Sex hormone concentrations and quality of life

Our study found a significant relationship between testosterone concentrations and QoL in the social relationship domain (D3) when testosterone measurements were taken within seven days of QoL assessment. This suggests that higher testosterone concentrations may be associated with improved social functioning, potentially by enhancing confidence, assertiveness, and social engagement – effects that have been observed in previous research on testosterone’s role in mood and behaviour (Hermans *et al*., 2006; Giltay *et al*., 2008; Miller *et al*., 2009).

This finding aligns with studies in both transgender and cisgender populations, where testosterone has been linked to increased sociability, reduced social anxiety, and greater emotional resilience (Rohr, 2002; Nguyen *et al*., 2018). One possible explanation is that testosterone modulates neurotransmitter systems, particularly dopamine and serotonin, which play key roles in motivation, reward processing, and social bonding (Barth *et al*., 2015).

However, the lack of a consistent relationship across longer intervals (e.g., up to one month) suggests that these effects of testosterone on QoL may be transient or influenced by fluctuations in hormone concentrations. Testosterone concentrations can vary depending on the timing of administration, metabolism, and receptor sensitivity, which could explain why the association was only evident in the shorter interval analysis. Additionally, this finding highlights the importance of considering short-term versus long-term hormonal effects in future research, as acute changes in testosterone may have more immediate effects on mood and behaviour than chronic baseline levels.

Importantly, our findings do not suggest that testosterone alone determines social well-being. While hormonal influences on self-confidence, energy levels, and social motivation may play a role, external factors such as social support, gender affirmation, and societal acceptance remain critical determinants of QoL (Wilson *et al*., 2016; Verbeek *et al*., 2020). The reciprocal interactions between biological, psychological and social factors warrant further investigation in longitudinal studies, ideally with controlled hormone administration and repeated assessments of QoL over time.

Our study did not find a linear relationship between oestradiol concentrations and QoL, which could be due to multiple effects of oestradiol. On the one hand, feminising GAHT facilitates the transitioning process by inducing physical changes, which may enhance certain aspects of QoL (T’Sjoen *et al*., 2019). On the other hand, the potential negative effects of oestradiol on the mental wellbeing of TGD individuals could also influence the QoL of individuals AMAB (Giltay *et al*., 2008). The finding from our study is consistent with a study by Ginger et al., from 2023, which also found no relationship between oestradiol concentrations and wellbeing (Ginger *et al*., 2023). This could reflect that there is no relationship between oestradiol and QoL, and hence that it is not (solely) the oestradiol concentration that matters. Furthermore, future studies with higher evidence and a larger sample are needed to further explore this, while taking into consideration other concurrent factors that might impact QoL in TGD individuals AMAB which GAHT might not be able to offset. Prejudice, discrimination, rejection, victimisation and non-affirmation of gender identity are experiences to which transgender women and transfeminine individuals can be particularly exposed and which impact well-being and QoL (Wilson *et al*., 2016; Arayasirikul *et al*., 2022).

### Strengths and limitations

Our study utilised a combination of subjective and objective data from the TraCK, which included self-reported responses from the WHOQOL-BREF questionnaire and blood tests of oestradiol and testosterone. This comprehensive approach ensured that the study had access to high-quality data from a diverse cohort encompassing individuals with diverse gender identities and undergoing various gender-affirming treatments. By investigating the difference in QoL between TGD individuals receiving GAHT and controls, our study contributes new insights into the knowledge gap of existing evidence within this field. Additionally, our study also compared QoL between individuals AMAB and individuals AFAB, which contributes to an understanding of gender-specific differences in QoL. Furthermore, this study provides valuable insights into the relationship between physiological measurements such as sex hormone concentrations and QoL for which the evidence is still limited. To measure QoL four domain scores were calculated and analysed instead of a total QoL score, which provided a more comprehensive understanding of different aspects of QoL.

However, our study also has some limitations. We used a cross-sectional study design dividing the relatively large sample of 360 TGD individuals into four smaller subgroups. These small subgroups set a limitation for the numbers of sub- and confounder-adjusted analyses. However, future studies could benefit from considering both gender-affirming surgery and social transition, as these factors may influence the quality of life (QoL) of TGD individuals. Because the WHOQOL-BREF relies on subjective interpretation of the questions, the internal validity of the questionnaire might have been affected, as participants could have interpreted certain questions differently. Furthermore, this study has only investigated the relationship between GAHT and QoL and has not taken into account other factors that might significantly impact QoL. Among these, previous or current psychiatric diagnoses e.g. depression and anxiety have not been considered, which would have been a major advantage as it is known that mental health conditions can influence QoL and thereby the results of our study. Another limitation was the presence of missing data in questionnaire items for some participants However, this was handled in the analysis by imputation. Regarding the blood tests of sex hormones, a limitation was the inability to obtain these samples precisely at the same time as the completion of the questionnaire responses. Consequently, there was a maximum time difference of up to one month between the two measurements. In addition, both the time of day for blood sampling and the time between administering GAHT and the blood sampling could be a limitation of this study, as concentraions of sex hormones can change throughout the day and rise significantly after the administration of GAHT. Furthermore, it can be discussed if the inclusion criterion regarding having received GAHT for at least three months for participants in the GAHT group is sufficient. The three months were chosen because the first GAHT-induced changes are developed at this point. However, other secondary sex characteristics might first be fully developed after 6, 12 or 24 months (Defreyne *et al*., 2023).

### Future perspectives

Given the variability in QoL outcomes following GAHT, a precision health approach – tailoring medical treatments to an individual’s genetics, lifestyle, and environmental factors – may help optimise therapeutic outcomes for TGD individuals (Sabatello *et al*., 2023). Incorporating a bio-psycho-social perspective of transition is also essential, recognising that QoL and well-being is influenced not only by biological factors but also by psychological processes and social contexts. In addition, factors such as gender minority stressors, resilience, and intersectionality are likely to shape individual responses to treatment and should be systematically incorporated into both clinical care and future research.

## Conclusion

In conclusion, this study found that QoL was significantly higher among TGD individuals receiving GAHT compared to those who had not initiated treatment, with the greatest improvements observed in the psychological health domain (D2). Additionally, a statistically significantly higher QoL was found for individuals AFAB compared to individuals AMAB receiving GAHT which was primarily driven by the social domain. A significant relationship between testosterone concentrations and the social domain of QoL emerged when measurements were taken within a seven-day interval. However, no consistent linear relationship between sex hormone concentrations and QoL was found overall.

With these findings, this study provides insights into how GAHT impacts the QoL of TGD individuals, – particularly psychological and social dimensions – while also highlighting the complex interplay of biological, psychological, and social factors. This contributes new knowledge and can thereby create more attention for evidence-based treatment of TGD individuals, which can significantly improve their well-being and healthcare outcomes.

## Data Availability

All participants provided written informed consent prior to participation, and all procedures were conducted in accordance with the Declaration of Helsinki. Participant confidentiality was maintained, and data were anonymized to ensure privacy. The study was registered by the North Jutland Region (no. F2024-012).
